# The relationship of insulin resistance estimated by triglyceride glucose index and coronary plaque characteristics

**DOI:** 10.1097/MD.0000000000010726

**Published:** 2018-05-25

**Authors:** Ki-Bum Won, Yun Seok Kim, Byoung Kwon Lee, Ran Heo, Donghee Han, Ji Hyun Lee, Sang-Eun Lee, Ji Min Sung, Iksung Cho, Hyung-Bok Park, In-Jeong Cho, Hyuk-Jae Chang

**Affiliations:** a Division of Cardiology, Ulsan University Hospital; b Division of Thoracic and Cardiovascular Surgery, Ulsan University Hospital, University of Ulsan College of Medicine, Ulsan; c Division of Cardiology, Gangnam Severance Hospital, Yonsei University College of Medicine; d Division of Cardiology, Hanyang University Seoul Hospital, Hanyang University College of Medicine; e Division of Cardiology, Severance Cardiovascular Hospital, Yonsei University College of Medicine, Yonsei University Health System; f Division of Cardiology, Chung-Ang University Hospital; g Division of Cardiology, Catholic Kwandong University International St. Mary's Hospital, Incheon, South Korea.

**Keywords:** coronary artery disease, coronary calcification, insulin resistance

## Abstract

The triglyceride glucose (TyG) index is a useful surrogate marker for insulin resistance, which is an important risk factor for coronary artery disease (CAD). However, data on the relationship of the TyG index and coronary plaque characteristics are limited.

This study included 2840 participants with near-normal renal function who underwent coronary computed tomography angiography. CAD was defined as the presence of any plaques, and obstructive CAD was defined as the presence of plaques with ≥50% stenosis. The relationship between the TyG index and noncalcified plaque (NCP), calcified or mixed plaque (CMP), and coronary artery calcium score (CACS) was evaluated.

All participants were stratified into 4 groups based on the quartiles of the TyG index. The prevalence of CAD and obstructive CAD significantly increased with increasing quartiles. The risk for NCP and obstructive NCP was not different among all groups. However, compared with group I (lowest quartile), the risk for CMP was higher in groups III (odds ratio [OR]: 1.438) and IV (highest quartile) (OR: 1.895) (*P* < .05), and that for obstructive CMP was higher in groups II (OR: 1.469), III (OR: 1.595), and IV (OR: 2.168) (*P* < .05). Multivariate regression analysis showed that the TyG index was associated with an increased risk for CAD (OR: 1.700), obstructive CAD (OR: 1.692), and CACS >400 (OR: 1.448) (*P* < .05).

The TyG index was independently associated with the presence and severity of CAD due to an increased risk for CMP.

## 1 Introduction

Coronary artery disease (CAD) is a major cause of morbidity and mortality worldwide.^[1]^ Insulin resistance (IR) has been demonstrated to play a substantial role in the progression of coronary atherosclerosis.^[2,3]^ However, data on the relationship between IR and coronary plaque characteristics, particularly in a relatively healthy adult population, are limited. Recently, the triglyceride glucose (TyG) index has been used in clinical practice as a simple and reliable surrogate marker of IR.^[4–7]^

Coronary artery calcium score (CACS) is traditionally regarded as a good marker of coronary atherosclerosis because it represents the degree of atheromatous plaque burden.^[8,9]^ With the advent of imaging technology, coronary computed tomography angiography (CCTA) has become a noninvasive tool for evaluating coronary atherosclerosis with a high diagnostic accuracy for detecting coronary plaques.^[10–12]^ Thus, in the present study, we evaluated the relationships between IR, as assessed by the TyG index, and the presence and severity of CAD and coronary plaque characteristics.

## 2 Methods

### 2.1 Subjects and study design

This observational, retrospective study included 2840 consecutive subjects with near-normal renal function (estimated glomerular filtration rate [GFR]: ≥60 mL/min/1.73 m^2^) who underwent CCTA in a self-referral setting during a general health check-up between January 2004 and April 2009. Subjects were excluded from this study if they met one of the following criteria: age <35 years; clinical history of cardiovascular disease, cerebrovascular disease, or malignancy; estimated GFR <60 mL/min/1.73 m^2^; or insufficient medical record for TyG index calculation. The participant's medical history on hypertension, diabetes, and dyslipidemia, and smoking status were systematically collected. All blood samples were obtained after a minimum fasting time of 8 hours and were analyzed for lipid profile, including triglyceride, high- (HDL) and low-density lipoprotein (LDL) cholesterol, and glucose levels. The TyG index was calculated as ln (fasting triglycerides [mg/dL] × fasting glucose [mg/dL]/2).^[4]^ The body mass index was calculated as weight (kg)/height (m^2^). The kidney function was assessed by estimated GFR using the formula validated in the Modification of Diet in Renal Disease Study.^[13]^ Hypertension was defined as systolic and diastolic blood pressures (BPs) of at least 140 and 90 mm Hg, respectively, or those taking antihypertensive medications. Diabetes was defined as either a fasting glucose level of ≥126 mg/dL, a referral diagnosis of diabetes, or undergoing antidiabetic treatment. Dyslipidemia was defined as a total cholesterol level of ≥240 mg/dL, LDL level of ≥130 mg/dL, HDL level of ≤40 mg/dL, triglyceride level of ≥150 mg/dL, or taking lipid-lowering medications. A participant was considered a current smoker if he/she consistently smoked or smoked within 1 month before the study. The study protocol was approved by the institution's ethics committee. Because of the retrospective nature of this study, the institutional review board waived the need for written informed consent from study participants.

### 2.2 Coronary computed tomography angiography protocol

Subjects with an initial heart rate of at least 65 beats/min before undergoing computed tomography (CT) scan were administered a single oral dose of 50 mg metoprolol tartrate (Betaloc; Yuhan, Seoul, South Korea) 1 hour before CT scan, unless the administration of β-blockers was contraindicated. Imaging was performed for all patients using a 64-slice CT scanner (Sensation 64; Siemens Medical System, Forchheim, Germany). Initially, a nonenhanced prospective electrocardiogram (ECG)-gated scan to evaluate CACS was performed with the following parameters: rotation time of 330 ms, slice collimation of 0.6 mm, slice width of 3.0 mm, tube voltage of 100 to 120 kV, tube current of 50 mA, and table feed/scan of 18 mm. Subsequently, CCTA was performed using retrospective ECG gating with the following scan parameters: rotation time of 330 ms, slice collimation of 64 × 0.6 mm, tube voltage of 100 to 120 kV, tube current of 400 to 800 mA (depending on the patient size), table feed/scan of 3.8 mm, and pitch factor of 0.2. ECG-based tube current modulation was applied to 65% of the R–R interval. A real-time bolus-tracking technique was applied to trigger scan initiation. Contrast enhancement was achieved with 60 mL iopamidol (370 mg/mL iodine, Iopamiro; Bracco, Milan, Italy) injected at 5 mL/s, followed by injection of 30 mL diluted contrast medium (saline–contrast agent ratio of 7:3), and then 30 mL saline at 5 mL/s with a power injector (Envision CT; Medrad, Indianola, PA) through the antecubital vein. The image was reconstructed using a commercially available software (Wizard; Siemens Medical Solutions) in the scanner workstation. The axial images were reconstructed retrospectively at 65% of the R–R interval for each cardiac cycle. Additional data sets were obtained for various points of the cardiac cycle if artifacts were present, and the data set with the minimum artifact was selected for further analysis. The reconstructed image data sets were transferred to an offline workstation (Aquarius Workstation; TeraRecon Inc., San Mateo, CA). Each lesion identified was examined using maximum-intensity projection and multiplanar reconstruction techniques on a short axis and along multiple longitudinal axes. The lesions were classified by the maximal stenosis of luminal diameter observed on any plane.

### 2.3 Measurement of coronary plaque and calcium

All CCTA images were evaluated by 2 experienced cardiac radiologists. In cases of disagreement, a joint reading was performed to reach a consensus. Plaques were defined as structures >1 mm^2^ within and/or adjacent to the vessel lumen that were clearly distinguishable from the lumen and surrounding pericardial tissue. Obstructive plaques were defined as plaques with ≥50% luminal stenosis. CAD was defined by the presence of any plaques, and obstructive CAD was defined by the presence of obstructive plaques. All plaques were classified into 2 subtypes based on the presence of coronary calcification: noncalcified plaque (NCP) and calcified or mixed plaque (CMP). CACS was evaluated based on the scoring system by using a previously described method.^[14]^ Severe coronary calcification was defined as CACS >400.

### 2.4 Statistical analysis

Continuous variables were expressed as mean ± standard deviation. Categorical variables were presented as absolute values and proportions. To compare the characteristics among the TyG index groups, one-way analysis of variance was used for continuous variables, and the χ^2^ test or Fisher exact test was used for categorical variables, as appropriate. Univariate logistic regression analysis was performed to identify the risk of coronary atherosclerotic parameters. Multivariate logistic regression analysis was used to identify the independent determinants for CAD, obstructive CAD, and CACS >400. The forced entry method was used to enter independent variables into the multivariate regression analysis. All statistical analyses were performed using the Statistical Package for the Social Sciences version 19 (SPSS, Chicago, IL), and *P* < .05 was considered significant for all analyses.

## 3 Results

### 3.1 Clinical characteristics of subjects

Table 1 shows the clinical characteristics of the study participants. All participants were stratified into 4 groups based on their TyG index levels. Systolic and diastolic BP, body mass index, and levels of total cholesterol, triglyceride, HDL, LDL, fasting glucose, and creatinine were significantly different among the groups. No significant difference was found in age, levels of estimated GFR, incidence of hypertension, or smoking status.

**Table 1:**
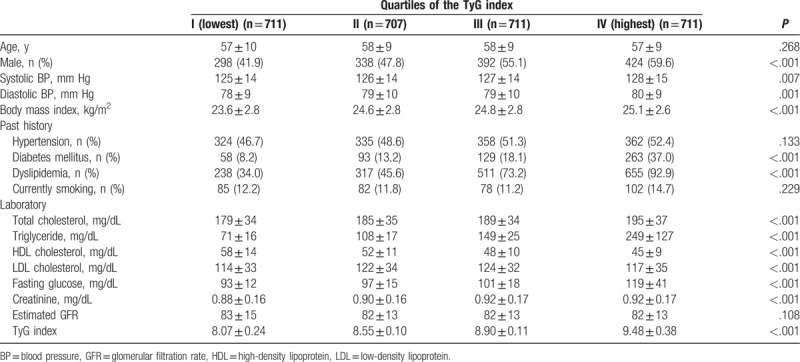
Baseline characteristics.

### 3.2 Coronary atherosclerosis based on triglyceride glucose quartile groups

Table 2 shows the incidence of coronary atherosclerotic parameters based on the TyG quartile groups. The incidence of CAD and obstructive CAD significantly increased with increasing TyG index quartile groups. In addition, categorical CACS was significantly different among these groups. No significant difference was found in the incidence of NCP (group I [lowest quartile]: 6.5%; group II: 7.6%; group III: 8.9%; group IV [highest quartile]: 9.3%; *P* = .200) or obstructive NCP (group I: 2.7%; group II: 1.3%; group III: 2.0%; group IV: 3.1%; *P* = .103) in each group. However, the incidence of CMP (group I: 31.4%; group II: 35.8%; group III: 39.7%; group IV: 46.4%; *P* < .001) and obstructive CMP (group I: 8.7%; group II: 12.3%; group III: 13.2%; group IV: 17.2%; *P* ≤ .001) was different among these groups.

**Table 2:**
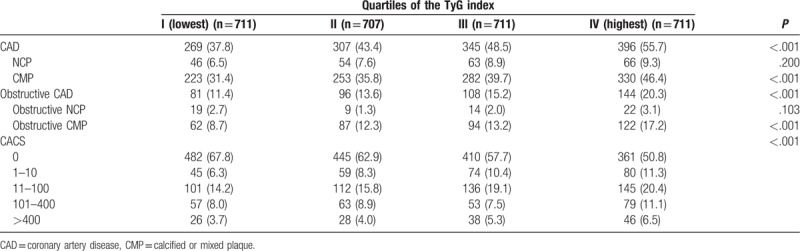
Coronary characteristics.

### 3.3 Association between the triglyceride glucose index and coronary atherosclerosis

**Figure 1:**
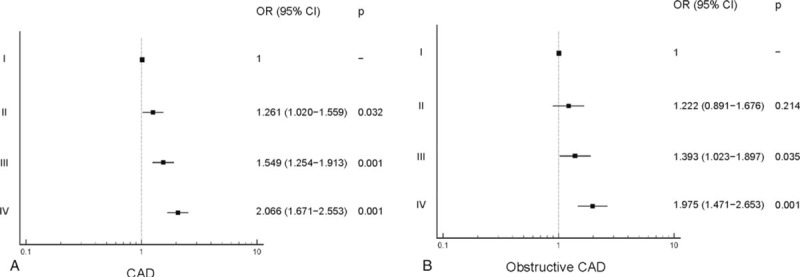
Risk of (A) CAD and (B) obstructive CAD based on the triglyceride glucose (TyG) index group. CAD = coronary artery disease, CI = confidence interval, OR = odds ratio.

**Figure 2:**
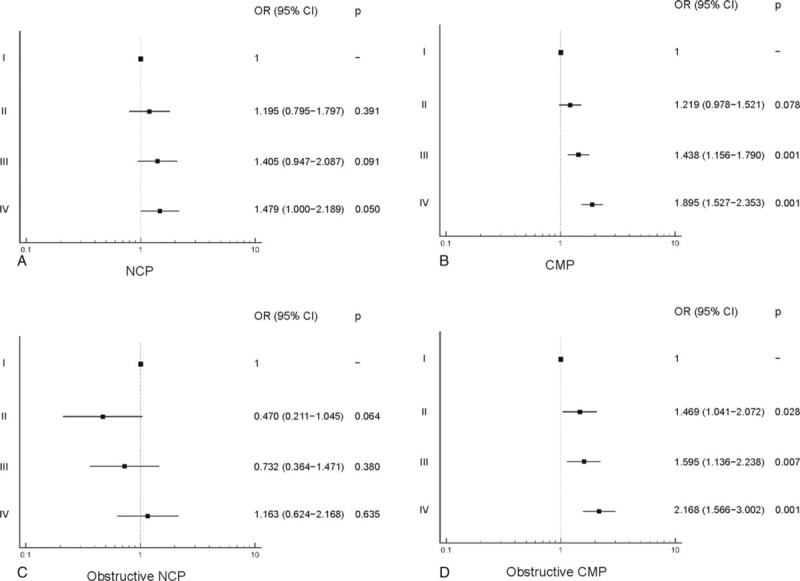
Relationship of the triglyceride glucose (TyG) index and subtypes of coronary plaque as (A) NCP, (B) CMP, (C) obstructive NCP, and (D) obstructive CMP. CI = confidence interval, CMP = calcified or mixed plaque, NCP = noncalcified plaque, OR = odds ratio.

Compared with group I, the risk for CAD was higher in groups II (odds ratio [OR]: 1.261; 95% confidence interval [CI]: 1.020 − 1.559; *P* = .032), III (OR: 1.549; 95% CI: 1.254−1.913; *P* = .001) and IV (OR: 2.06; 95% CI: 1.671−2.553, *P* = .001), and that of obstructive CAD was higher in groups III (OR: 1.393; 95% CI: 1.023−1.897; *P* = .035) and IV (OR: 1.975; 95% CI: 1.471−2.653; *P* = .001) (Fig. 1). The risk for developing NCP and obstructive NCP was not significantly different among the groups. However, compared with group I, the risk for CMP was higher in groups III (OR: 1.438; 95% CI: 1.156−1.790; *P* = .001) and IV (OR: 1.895; 95% CI: 1.527−2.353; *P* = .001), and that of obstructive CMP was higher in groups II (OR: 1.469; 95% CI: 1.041−2.072; *P* = .028), III (OR: 1.595, 95% CI: 1.136−2.238; *P* = .007), and IV (OR: 2.168, 95% CI: 1.566−3.002; *P* = .001) (Fig. 2).

### 3.4 Impact of clinical variables on coronary atherosclerosis

Table 3 shows the results of the multivariate regression analysis to identify the relationship between traditional metabolic risk factors and the presence and severity of CAD and severe coronary calcification. Age (OR: 1.099; 95% CI: 1.087–1.112; *P* < .001), male sex (OR: 2.722; 95% CI: 2.252–3.289; *P* < .001), body mass index (OR: 1.230; 95% CI: 1.030–1.470; *P* = .022), and TyG index (OR: 1.700; 95% CI: 1.436–2.013; *P* < .001) were associated with an increased risk for CAD. In addition, age (OR: 1.067; 95% CI: 1.052–1.081; *P* < .001), male sex (OR: 2.375; 95% CI: 1.843–3.060; *P* < .001), and TyG index (OR: 1.692; 95% CI: 1.371–2.088; *P* < .001) were associated with an increased risk for obstructive CAD. Furthermore, age (OR: 1.101; 95% CI: 1.074–1.128; *P* < .001), male sex (OR: 2.204; 95% CI: 1.437–3.380; *P* < .001), increased BP (OR: 1.699; 95% CI: 1.027–2.810; *P* = .039), LDL >130 mg/dL (OR: 0.598; 95% CI: 0.378–0.946; *P* = .028), and TyG index (OR: 1.448; 95% CI: 1.028–2.039; *P* = .034) were associated with CACS >400.

**Table 3:**
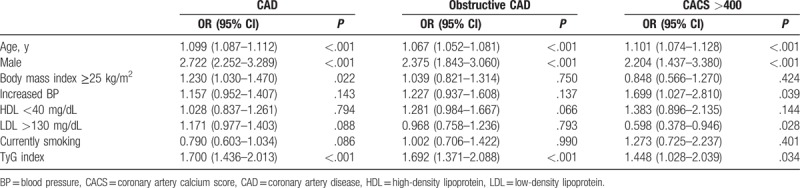
Association between clinical variables and coronary atherosclerotic parameters.

## 4 Discussion

The primary findings of the present study were that the TyG index was associated with the presence of CAD, obstructive CAD, and severe coronary calcification after adjusting for confounding factors. These results were related to the increased risk for developing CMP and obstructive CMP with increasing TyG index.

IR has been well known to play a pivotal role for metabolic disorders.^[15]^ The homeostatic model assessment of IR (HOMA-IR) has been traditionally used to measure IR.^[16,17]^ However, the measurement of insulin levels is necessary for calculating the HOMA-IR. Because insulin levels are not usually measured during general health check-up in South Korea, calculating the HOMA-IR to estimate IR is an inconvenient method for this population. Recently, several studies reported that the TyG index is closely correlated with the HOMA-IR.^[18,19]^ Moreover, some studies showed that the predictive value of the TyG index for IR is better than that of the HOMA-IR.^[6,20]^ Thus, the TyG index is being considered as a simple surrogate marker of IR for clinical practice.

A number of studies have suggested the significance of IR in the development of CAD. Hanley et al^[21]^ reported an independent association between the HOMA-IR and risk for CAD in the San Antonio Heart Study. The Bruneck study also revealed that IR is associated with subsequent symptomatic CAD independent of traditional risk factors in the general population.^[2]^ Eddy et al^[3]^ emphasized that IR is the single most common cause of CAD by using the Archimedes model of diabetic dyslipidemia. However, data on the relationship between IR and coronary atherosclerosis characteristics in the general population are scarce. Recently, Kim et al^[22]^ reported that the TyG index was independently associated with CACS in a healthy adult population. Interestingly, they identified that the TyG index was more independently associated with the presence of coronary artery calcium than the HOMA-IR. However, this study was limited because only CACS was used as a coronary atherosclerotic parameter.

CCTA is a useful and noninvasive tool for the evaluation of coronary atherosclerosis. In the present study, we identified that the TyG index is independently associated with the presence and severity of CAD and severe coronary calcification by using CCTA. One result worth noting is that the risk for developing NCP and obstructive NCP was not significantly affected by the TyG index. Considering the substantial impact of diabetes on coronary calcification, these results may be associated with the increased prevalence of diabetes with increasing TyG index.^[23]^ One important mechanism might be hyperglycemic damage which activates endothelial dysfunction and vascular inflammation. Also, hyperglycemia increases oxidative stress by stimulating glucose oxidation.^[24]^ A previous observational study reported that CACS could be an effective gatekeeper to evaluate obstructive CAD by using CCTA in asymptomatic subjects with established diabetes because obstructive NCP was hardly observed in those subjects.^[25]^ The results of the present study were consistent with those of previous investigations and may imply that IR strongly influences the progression of coronary plaque with concomitant coronary calcification.

The present study has some limitations. First, it used a retrospective design based on the healthy population who underwent a general health check-up. Thus, the results may have been influenced by unobserved confounders or potential selection biases. Second, we could not eliminate the possible effects of the medications on CAD because of the observational design. Third, we did not measure the HOMA-IR because the examination of insulin levels is not typically included in general health check-up at our institution. However, the close relationship between the TyG index and HOMA-IR was already well-established, as previously described. Finally, we did not investigate the association between the TyG index and adverse plaque characteristics, such as low plaque attenuation, positive remodeling, and spotty calcification.^[26,27]^ Further large-scale prospective investigations may be necessary to identify the impact of IR on coronary plaque vulnerability.

In conclusion, the TyG index is significantly associated with the presence and severity of CAD and severe coronary calcification after adjusting for confounding factors. These results are associated with the increased risk for CMP with increasing TyG index.

## Author contributions

**Conceptualization:** Ki-Bum Won, Byoung Kwon Lee, Ran Heo, Hyung-Bok Park, In-Jeong Cho, Hyuk-Jae Chang.

**Data curation:** Hyuk-Jae Chang.

**Formal analysis:** Ki-Bum Won, Yun Seok Kim, Donghee Han, Ji Hyun Lee, Ji Min Sung.

**Investigation:** Ki-Bum Won, Donghee Han, Ji Hyun Lee, Sang-Eun Lee, Iksung Cho, In-Jeong Cho, Hyuk-Jae Chang.

**Methodology:** Ran Heo, Hyuk-Jae Chang.

**Supervision:** Byoung Kwon Lee, Ji Min Sung, Iksung Cho, Hyung-Bok Park, Hyuk-Jae Chang.

**Writing – original draft:** Ki-Bum Won, Yun Seok Kim.

**Writing – review and editing:** Hyuk-Jae Chang.
